# Comparison of morphological and functional parameters after switching therapy from brolucizumab to faricimab in neovascular age-related macular degeneration

**DOI:** 10.1186/s12886-026-05238-1

**Published:** 2026-08-14

**Authors:** Agnes Boltz, Jasmin Paster, Sarah Mehany, Veronika Pia Vécsei-Marlovits

**Affiliations:** 1 Department of Ophthalmology, Hietzing Hospital, Wolkersbergenstraße 1, Vienna, 1130 Austria; 2 Karl-Landsteiner Institute for Process Optimization and Quality Management in Cataract Surgery, Vienna, Austria

**Keywords:** Macular neovascularization, Brolucizumab, Faricimab, Switch

## Abstract

**Background:**

Brolucizumab, an anti-vascular endothelial growth factor (VEGF) agent, offers extended dosing intervals in neovascular age-related macular degeneration (nAMD) but carries a rare risk of retinal vasculitis. Faricimab, a bispecific antibody targeting VEGF and angiopoietin-2 (Ang-2), maintained efficacy with dosing intervals of up to 16 weeks in the TENAYA and LUCERNE trials and carries a lower reported inflammatory risk. Patients may therefore be switched from brolucizumab to faricimab. This study examines the morphological and functional response after such a switch.

**Methods:**

Patients with nAMD were included in this retrospective case series. Eligibility required at least three consecutive brolucizumab injections before switching to faricimab. The primary outcome was the change in central retinal thickness (CRT); secondary outcomes were the change in best-corrected visual acuity (VA) and adverse events.

**Results:**

The study included 31 eyes from 28 patients treated with brolucizumab and switched to faricimab. Mean visual acuity (VA) was 0.3 logMAR (Snellen equivalent ~20/40) at visit -3, with no significant change after three brolucizumab injections (0.3 logMAR, ~20/40, p=0.932). After switching to faricimab, mean VA was 0.332 logMAR (~20/40) with no statistically significant difference from the value after three brolucizumab injections (p=0.187). Central retinal thickness (CRT) remained stable before (355.00µm to 347.29µm, p=0.538) and after switching (366.54µm, p=0.190). Of the 11 eyes that were free of fluid before the switch, 10 did not remain dry, and CRT increased significantly (282.9µm to 367.9µm, p=0.013) after extending intervals on average by +2.3 weeks (min 0 to max +5 weeks). However, visual function remained stable (p=0.559). In contrast, two eyes became dry with shorter intervals (-2 weeks), one eye remained dry (+2 weeks) and 18 eyes remained active (-0.5 weeks).

**Conclusions:**

Overall, morphological and functional parameters remained stable after switching to faricimab, and the safety profile was stable. Because every eye received both agents sequentially and no concurrent brolucizumab control group was included, this was not a head-to-head comparison. The increase in retinal thickness in some cases highlights the need for individualized treatment based on patient response.

## Background

Age-related macular degeneration (AMD) is a major cause of visual impairment and affects approximately 200 million people worldwide [1, 2], of whom 10–20% develop neovascular AMD (nAMD). This subgroup is characterized by the growth of abnormal vessels that leads to fluid accumulation and consequently to retinal damage. The introduction of anti-vascular endothelial growth factor (VEGF) antibodies has revolutionized the treatment of this disease entity [3–7]. The associated treatment burden, however, remains a challenge for patients and caregivers alike.

Brolucizumab (Beovu^®^, Novartis, Basel, Switzerland) is an anti-VEGF agent developed for the treatment of nAMD. Approved by the U.S. Food and Drug Administration (FDA) in 2019 and the European Medicines Agency (EMA) in 2020, brolucizumab emerged as a promising option for patients facing the burden of frequent intravitreal injections. Clinical trials, including the pivotal HAWK and HARRIER studies, demonstrated its efficacy in improving visual outcomes and in reducing retinal fluid, while offering the potential for injection intervals of up to 12 weeks [8]. Post-marketing surveillance, however, revealed rare but serious adverse events, including retinal vasculitis and retinal vascular occlusion [9], prompting the search for a viable therapeutic alternative.

Faricimab (Vabysmo^®^, F. Hoffmann-La Roche, Basel, Switzerland) is a bispecific antibody that targets both VEGF and angiopoietin-2 (Ang-2). The TENAYA and LUCERNE trials demonstrated that faricimab maintains efficacy with dosing intervals extended to up to 16 weeks [10]. Moreover, this dual mechanism is intended to address vascular destabilization and inflammation more comprehensively [11, 12].

Due to the more favorable safety profile, and because of the need for extended dosing intervals, patients receiving anti-VEGF therapy with brolucizumab were gradually switched to faricimab at our department. The present study aimed to evaluate the morphological and functional response to faricimab in nAMD patients who were last treated with brolucizumab, rather than to perform a head-to-head comparison between the two agents.

## Materials and methods

This study was conducted after approval from the Ethics Committee of the City of Vienna had been obtained and adhered to the tenets of the Declaration of Helsinki. This study was designed as a retrospective case series. The primary outcome measure was the change in central retinal thickness (CRT) after switching to faricimab; secondary outcome measures were the change in best-corrected visual acuity (VA), the proportion of eyes with subretinal fluid (SRF) and/or intraretinal fluid (IRF) at each visit, and the occurrence of ocular and systemic adverse events.

Patients with nAMD, confirmed by optical coherence tomography, optical-coherence tomography angiography or fluorescein angiography, were included. Other inclusion criteria were a history of anti-VEGF injections with at least three consecutive brolucizumab applications (visit − 3 to -1) directly prior to the first faricimab injection (visit 0). Among the exclusion criteria were presence of other retinal diseases, such as symptomatic epiretinal membrane, as well as other concomitant eye diseases that have significant impact on visual acuity (e.g. advanced glaucoma or cataract). Both eyes were included where both met these criteria, which applied to three patients. Macular neovascularization (MNV) subtype was classified at the time of study enrolment by means of optical coherence tomography, optical-coherence tomography angiography or fluorescein angiography, and was not taken over from the initial diagnosis.

Each control visit (from visit − 3 through visit 3) included best corrected visual acuity, slit-lamp examination and optical coherence tomography (Spectralis OCT, Heidelberg Engineering, Heidelberg, Germany). Best-corrected visual acuity was assessed as best-corrected Snellen visual acuity and converted to the logMAR scale for statistical analysis. Using the Spectralis Software, follow-up examinations were performed with a central grid containing 25 lines and retinal thickness was analyzed using the built-in ETDRS thickness map. If necessary, manual corrections of the retinal layer segmentation were undertaken by a trained physician. In addition, the presence of IRF and of SRF was assessed on the OCT volume scan at every visit by a trained physician, and each visit was assigned to one of four mutually exclusive categories: IRF only, SRF only, both SRF and IRF, or no fluid.

Injections were scheduled according to the treat and extend regimen, i.e. extension of intervals in the absence of intra-and/or subretinal fluid, new intraretinal or subretinal hemorrhage. Under this regimen, the treatment interval was extended in the absence of intra- and/or subretinal fluid or new haemorrhage and shortened when disease activity recurred, following the same scheme for brolucizumab and faricimab. Under the departmental standard operating procedure the extension applied in this situation was four weeks, and the longest interval recorded in this cohort was 16 weeks. Within this framework the decision in the individual eye remained with the treating physician, who could depart from the standard on clinical grounds. The interval to visit 1 therefore followed from the morphological findings at visit 0 together with this clinical judgement and was not fixed by protocol.

Data were analyzed using IBM SPSS statistics in the current version (28.0.1.0 for Windows). A general linear model with repeated measures as well as paired t-tests were performed to test for significance, which was assumed as *p* < 0.05. The general linear model was applied across all seven visits, whereas the paired t-tests were used for the following pairs of visits. For central retinal thickness, visit − 3 was compared with visit − 1 and with visit 0 for the brolucizumab phase, visit 0 with visit 1 for the effect of the first faricimab injection, and visit 0 with visit 3 for the effect of three faricimab injections. For best-corrected visual acuity, visit − 3 was compared with visit − 1 for the brolucizumab phase and visit − 1 with visit 1 for the effect of the first faricimab injection. In the subgroup analyses of eyes that were dry before the switch, and in the analysis of the ETDRS subfields, visit 0 was compared with visit 1 for both central retinal thickness and visual acuity. The proportions of eyes in the four fluid categories are reported descriptively for each visit. Poor responders and the remaining eyes were compared using the Mann–Whitney U test for continuous and Fisher’s exact test for categorical variables, and exact (Clopper–Pearson) 95% confidence intervals are given for proportions of adverse events.

## Results

In total, 31 eyes of 28 patients (22 female and 6 male) treated for nAMD with brolucizumab and switched to faricimab at our department were included in the study. The patients’ characteristics are shown in Table 1. All 31 eyes had previously received both bevacizumab and aflibercept, and 8 of the 31 eyes had additionally received ranibizumab, prior to brolucizumab and the subsequent switch to faricimab.

**Table 1:** Patients’ characteristics; SD = standard deviation; MNV = macular neovascularization

Age	81.21 years (SD 6.4 years)
Sex	female (24 eyes) / male (7 eyes)
Duration of treatment prior to switch	62.97 months (SD 48.07 months)
Number of injections prior to switch	34.48 (SD 19.97)
Prior treatment with bevacizumab	31 eyes
Prior treatment with aflibercept	31 eyes
Prior treatment with ranibizumab	8 eyes
MNV type 1 (occult)	7 eyes
MNV type 2 (classic)	24 eyes
MNV type 3 (RAP)	0 eyes

**Fig. 1:**
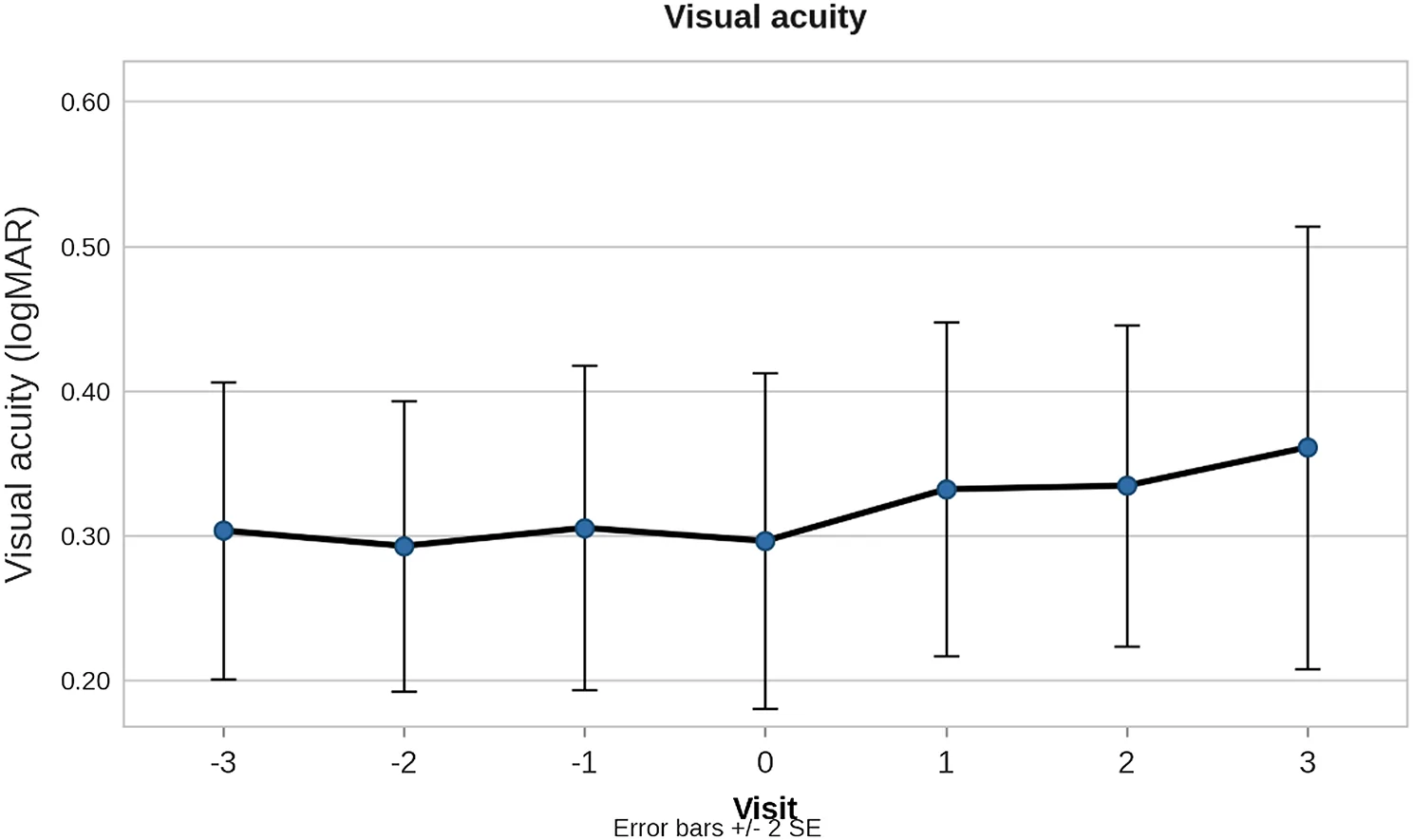
Visual acuity in logMAR. Means are shown with error bars. Visit 0 = switch to faricimab. Follow-up under faricimab is shown through visit 3 (i.e. after three faricimab injections). Error bars represent +/- 2 SE

Mean best-corrected visual acuity (VA) at visit − 3 was 0.3 logMAR (Snellen equivalent ~ 20/40) (standard deviation (SD) = 0.27) and did not significantly change after 3 injections with brolucizumab (0.3 logMAR, ~ 20/40; SD = 0.296; *p* = 0.932). After switching to a single dose of faricimab, the mean best-corrected visual acuity was 0.332 logMAR (~ 20/40) with no statistically significant difference from the value after 3 brolucizumab injections (*p* = 0.187; SD = 0.306; Fig. 1).

In this switched cohort, faricimab was administered at a median interval of 8 weeks (range 4 to 16 weeks) and brolucizumab at a median interval of 7 weeks (range 5 to 14 weeks). The magnitude of each adjustment was individualized rather than fixed; across the cohort the recorded interval changes ranged from a 2-week reduction to a 5-week extension (mean + 0.4 weeks), and in the poor-responder subgroup the interval was extended on average by 2.3 weeks (range 0 to 5 weeks). Of the nine eyes free of fluid at visit 0 the interval was extended in seven and left unchanged in two, and of the 22 eyes with persisting fluid it was shortened in nine, left unchanged in nine and extended in four. The mean interval between consecutive faricimab injections was 7.7 weeks (range 4.0 to 13.9 weeks) from visit 0 to visit 1, 7.2 weeks (range 4.0 to 10.0 weeks) from visit 1 to visit 2 and 8.0 weeks (range 3.9 to 16.1 weeks) from visit 2 to visit 3.

**Fig. 2:**
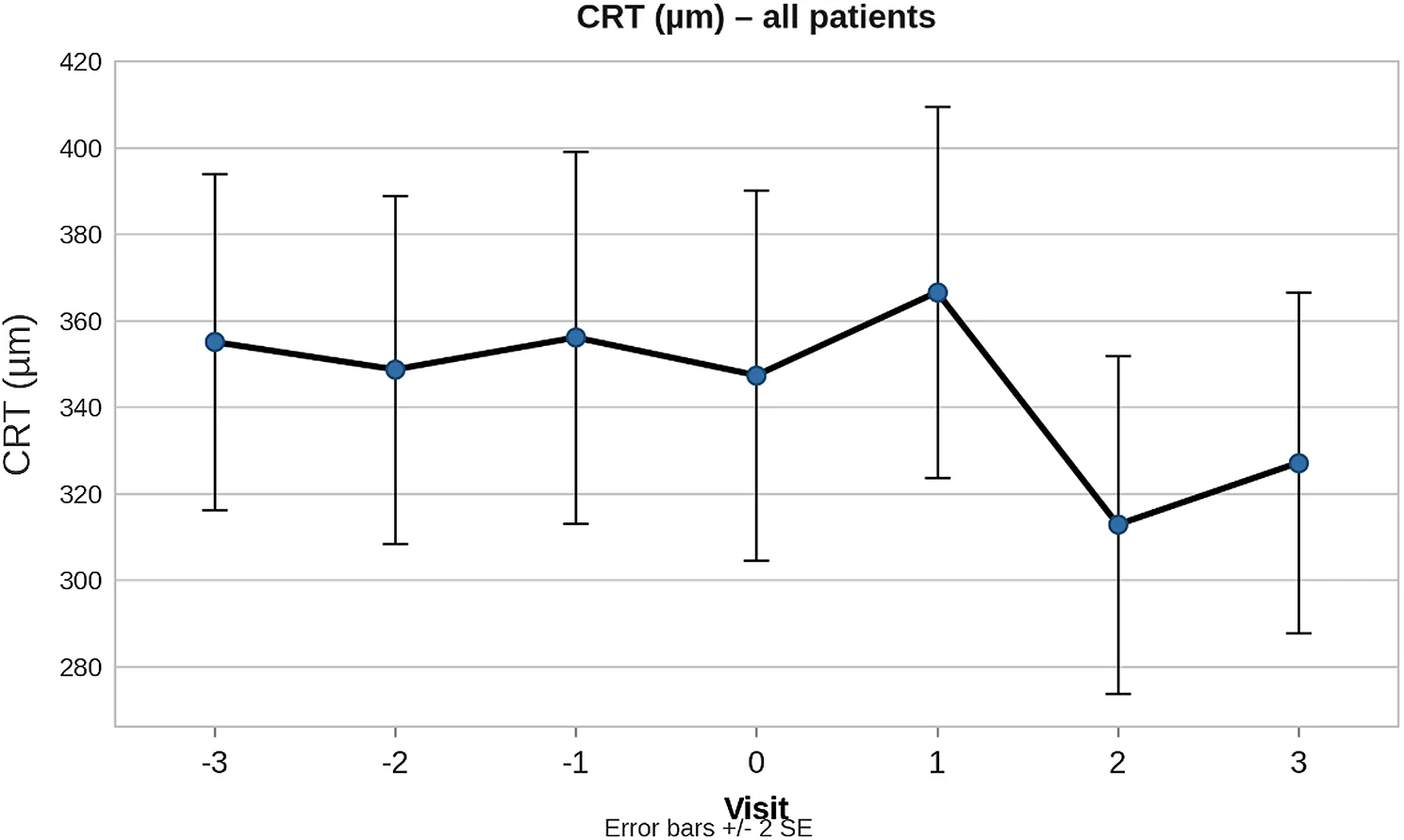
Central retinal thickness in micrometer. Means are shown with error bars of +/- 2 SE. Visit 0 = switch to faricimab. Follow-up under faricimab is shown through visit 3, i.e. after three faricimab injections

Similarly, central retinal thickness (CRT) remained stable prior to switching to faricimab, with mean CRT values of 355.00 μm (SD 103.00 μm) at visit − 3, 348.64 μm (SD 106.54 μm) at visit − 2, and 356.04 μm (SD 113.85 μm) at visit − 1 (visit − 3 vs. visit − 1: *p* = 0.949). After switching, CRT was 347.29 μm (SD 113.24 μm) at visit 0 and 366.54 μm (SD 113.67 μm) at visit 1 (*p* = 0.190). CRT was lowest at visit 2 with 312.75 μm (SD 103.36 μm) and was 327.07 μm (SD 104.36 μm) at visit 3, without a statistically significant difference from the switch visit (visit 0 vs. visit 3: *p* = 0.068), as shown in Fig. 2. On average, visit 1 took place 7.7 weeks after injection with faricimab (range 4.0–13.9 weeks). After a single dose of faricimab no effect was seen on the central 3-mm subfields surrounding the fovea. However, thickness was significantly increased in the nasal subfield of the central 6-mm subfield from 285.71 μm to 290.13 μm (*p* = 0.039).

**Fig. 3:**
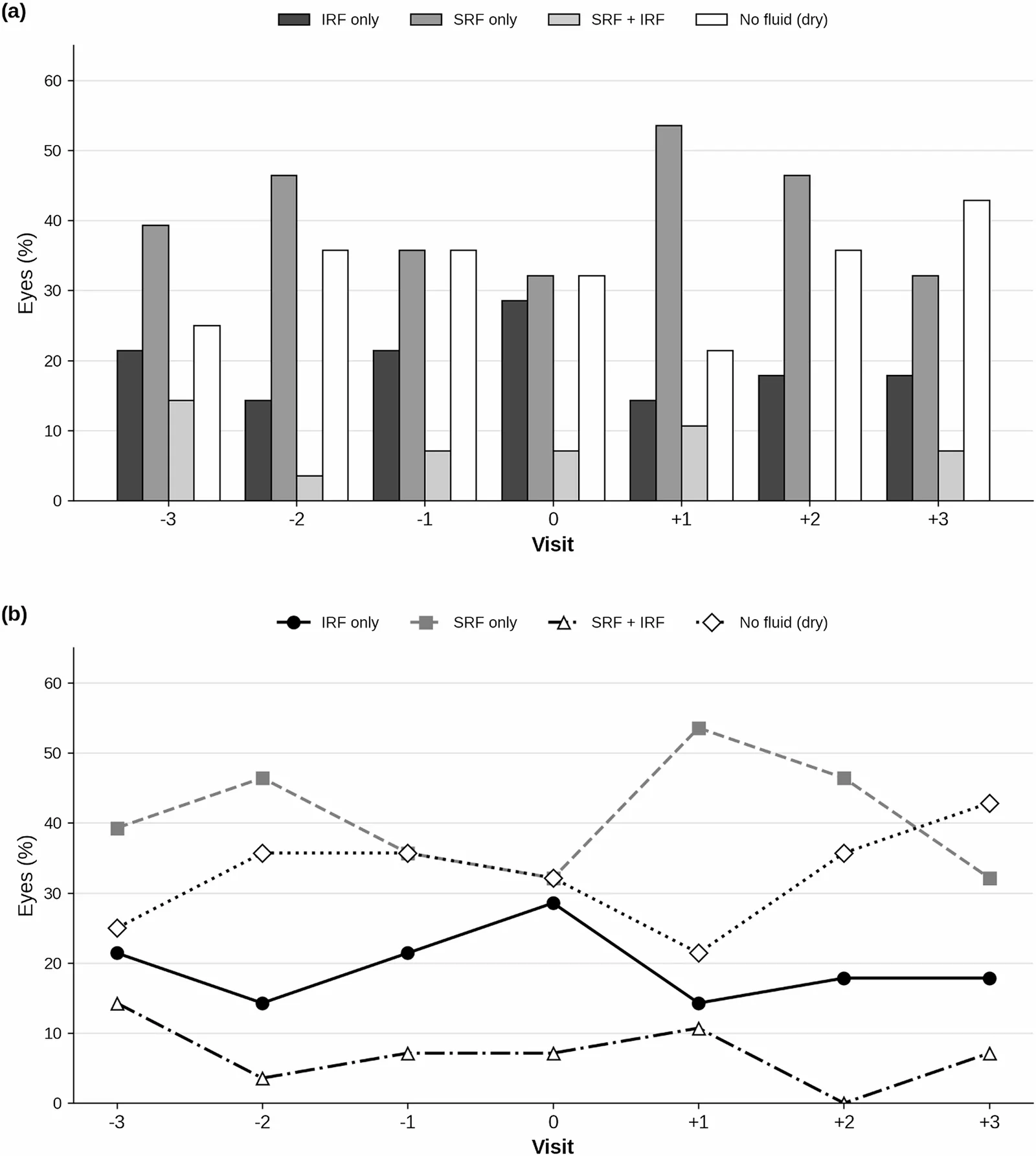
Distribution of macular fluid at each visit, at the same time points as Fig. 2. (**a**) Grouped bar chart. IRF = intraretinal fluid; SRF = subretinal fluid (**b**) The same data as trajectories over time; Visit 0 = switch to faricimab

The distribution of the four fluid categories at each visit is shown in Fig. 3. The proportion of fluid-free eyes was 25.0% (7/28) at visit − 3, 35.7% (10/28) at visit − 2, 35.7% (10/28) at visit − 1 and 32.1% (9/28) at visit 0. After the first faricimab injection it fell to 21.4% (6/28) at visit 1, while eyes with SRF only rose from 32.1% (9/28) to 53.6% (15/28). Over the following two injections the proportion of fluid-free eyes rose again to 35.7% (10/28) at visit 2 and 42.9% (12/28) at visit 3, the highest value of the observation period, and eyes with only SRF returned to 32.1% (9/28). Eyes with only IRF accounted for 14.3% (4/28) to 28.6% (8/28) and eyes with both SRF and IRF for 0% (0/28) to 14.3% (4/28) throughout, without a directional trend.

**Fig. 4:**
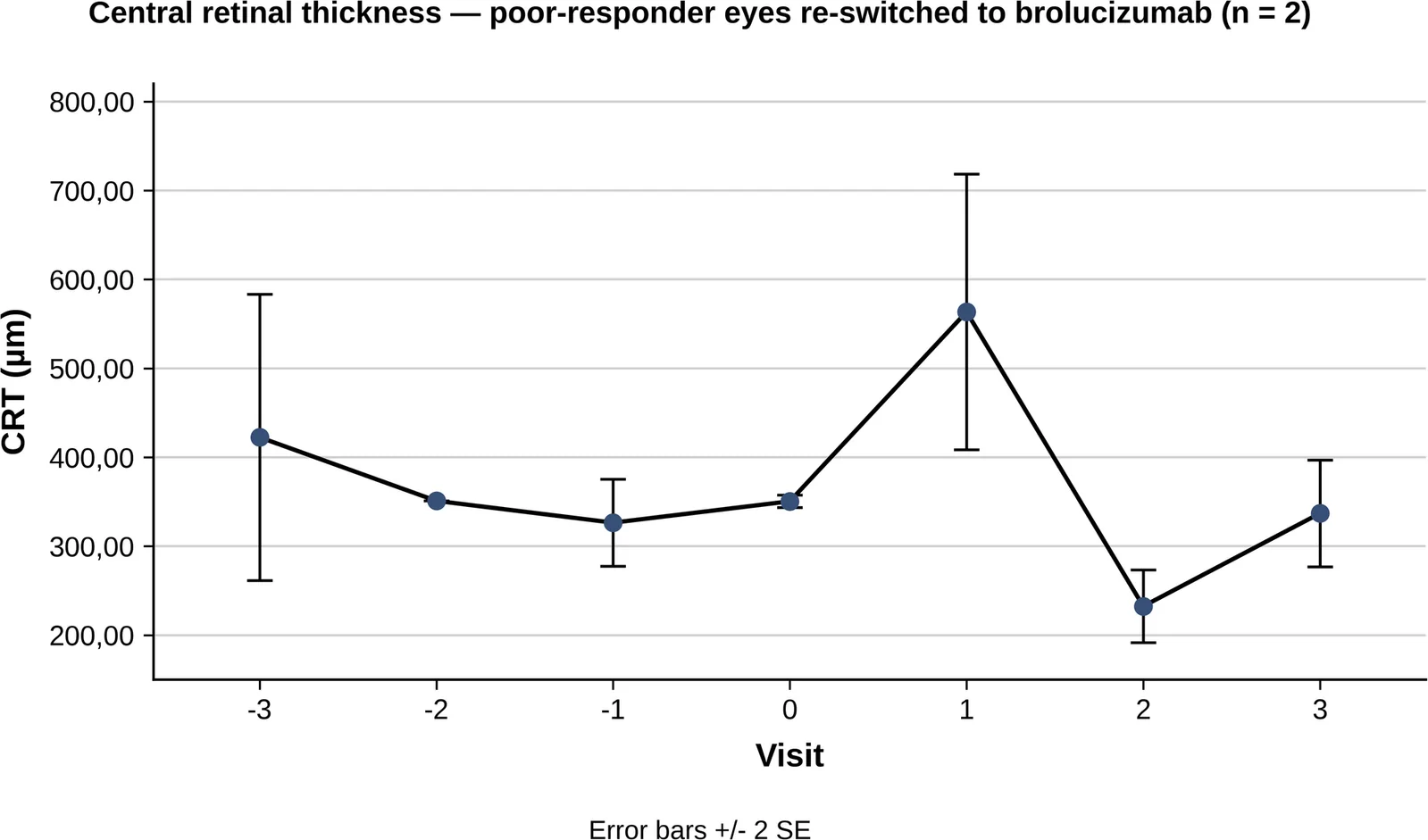
Central retinal thickness (micrometer) in the two poor-responder eyes that were re-switched to brolucizumab. Means are shown with error bars of +/- 2 SE. Visit 0 = switch to faricimab (first faricimab injection); visit 3 = after three faricimab injections. Follow-up under faricimab is shown through visit 3

**Fig. 5:**
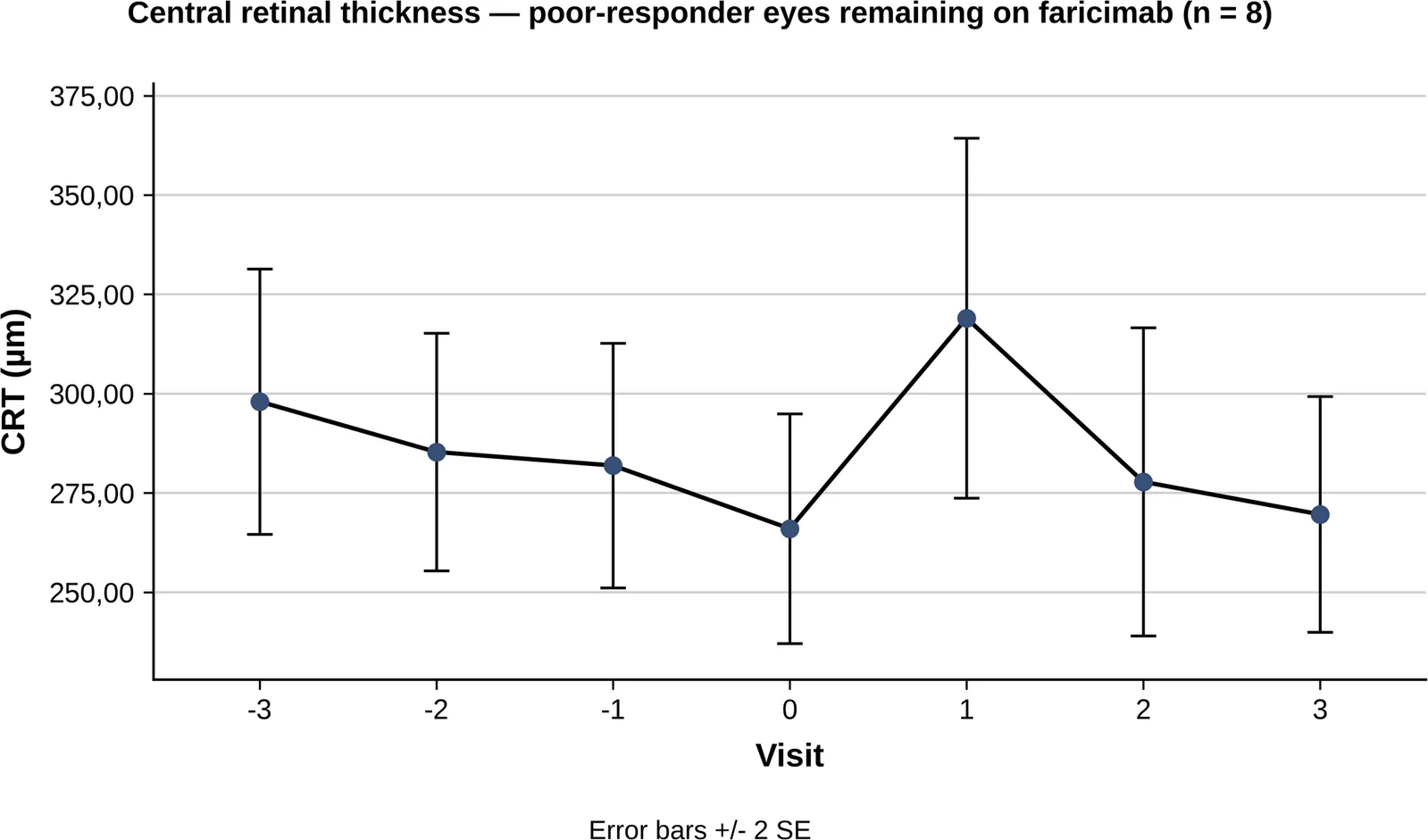
Central retinal thickness (micrometer) in the poor-responder eyes that remained on faricimab. Means are shown with error bars of +/- 2 SE. Visit 0 = switch to faricimab (first faricimab injection); visit 3 = after three faricimab injections. Follow-up under faricimab is shown through visit 3

Eleven eyes were dry prior to first treatment with faricimab. Of these, 10 eyes (32% of all eyes, 91% of the fluid-free eyes) did not remain dry after a single dose of faricimab. In this subgroup, the injection interval was extended on average by 2.3 weeks (min 0 weeks to max 5 weeks) and was accompanied by a significant increase in central retinal thickness from 282.9 μm (SD = 50.7 μm) to 367.9 μm (SD = 123.11 μm; *p* = 0.013, Figs. 4 and 5). This did not, however, translate into a significant loss of function, and visual acuity remained stable (0.2 to 0.23 logMAR (Snellen equivalent ~ 20/32 to ~ 20/34) (SD 0.16 and 0.2 respectively), *p* = 0.559).

In contrast, two eyes became dry after switching to faricimab and reducing the interval by 2 weeks whereas 18 eyes remained active (average reduction by -0.5 weeks). No serious adverse events were recorded up to the last available follow-up visit. No intraocular inflammation, retinal vasculitis, retinal vascular occlusion or endophthalmitis occurred during the observation period. Retinal pigment epithelium tears were observed in 2 eyes. No retinal detachment occurred during the observation period. Macular atrophy was observed in 12 eyes during the observation period.

After three faricimab injections, CRT remained largely stable at 327.07 μm (SD 104.36 μm) compared with 347.29 μm (SD 113.24 μm) at the switch visit (*p* = 0.068). All 10 poor-responder eyes had been free of fluid before the switch but developed renewed exudation after the first faricimab injection. Type 1 MNV was over-represented in this subgroup, accounting for 5 of the 10 poor-responder eyes (50%) compared with 2 of the 21 remaining eyes (about 10%); the subgroup comprised 5 eyes with Type 1 and 5 eyes with Type 2 MNV. After the renewed exudation, poor responders required shorter treatment intervals, and two eyes were switched back to brolucizumab, consistent with a higher overall treatment burden in this subgroup.

Baseline characteristics of the 10 poor-responder eyes and the remaining 21 eyes (all 31 eyes, as this comparison relies on baseline characteristics only) were compared to identify factors associated with a poor response. The over-representation of Type 1 MNV described above was the only statistically significant difference (5/10 versus 2/21; *p* = 0.022, Fisher’s exact test). Age at the switch did not differ (80.4 years, SD 9.1, versus 82.3 years, SD 4.2; *p* = 0.486), and all 10 poor-responder eyes were eyes of female patients. CRT at visit 0 was lower in poor responders (282.9 μm, SD 50.7, versus 365.7 μm, SD 124.5; *p* = 0.054) and their subsequent interval change was larger (+ 2.3 weeks, SD 1.7, versus − 0.6 weeks, SD 1.3; *p* < 0.001), but both differences follow from the definition of the subgroup: poor responders were by definition free of fluid at visit 0 and were therefore the eyes for which the treat-and-extend rule prescribed an extension. With 10 events no multivariable model was fitted and these comparisons are exploratory.

**Fig. 6:**
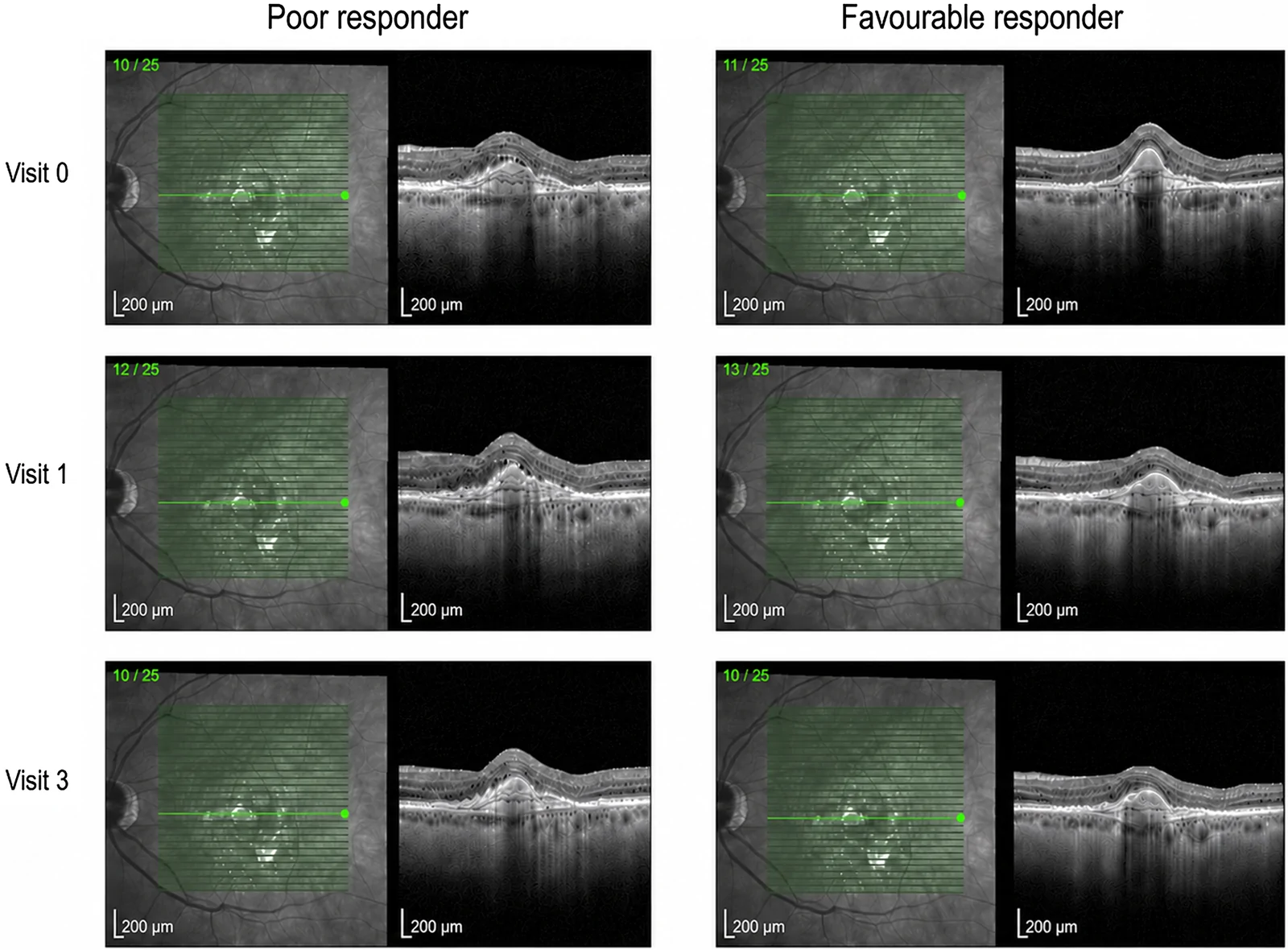
Representative cases. OCT scans over time for a representative favourable responder (a stable, fluid-free macula) and a representative poor responder

Two of the ten poor-responder eyes were switched back to brolucizumab after the renewed exudation. In these two eyes mean central retinal thickness rose markedly (350.5 μm at visit 0 to 563.5 μm at visit 1) before decreasing again (Fig. 4); because only two eyes were involved, these values are descriptive and were not tested statistically. In the remaining eight eyes, which continued faricimab, the post-switch increase was more modest (266.0 μm to 319.0 μm, *p* = 0.015) and likewise resolved over the subsequent visits (269.6 μm at visit 3) (Fig. 5). Individual eyes showed distinct morphological courses over the follow-up period; representative OCT images of a favourable and of a poor responder are shown in Fig. 6.

## Discussion

The present study provides real-world data on short-term anatomical and functional outcomes after switching from brolucizumab to faricimab in eyes with nAMD in a Caucasian population. Thirty-one eyes previously treated with at least three brolucizumab injections were analyzed. Over three faricimab injections, CRT and VA remained stable. In our cohort, a single dose of faricimab did not appear to provide greater durability, since 91% of the previously inactive lesions became exudative again once the interval had been extended by an average of 2.3 weeks (ranging from 0 to 5 weeks). However, after subsequent injections with faricimab, the renewed exudation regressed again in most of these eyes: in the eight eyes that continued faricimab, the post-switch increase resolved over the following visits (266.0 μm to 319.0 μm, then declining; Fig. 5), whereas the two eyes switched back to brolucizumab behaved differently (Fig. 4). The switch back in these two eyes also represents a limitation of our study, since it shortened the available follow-up. In this subgroup, VA nevertheless remained stable.

In the multicenter TRUCKEE study cohort, which included a subset of 56 eyes previously treated with brolucizumab, switching to faricimab led to a significant reduction in retinal thickness after one injection, whereas this reduction was no longer significant after 3 injections, and VA remained stable throughout follow-up [13].

A retrospective study comparing treatment-naïve patients with nAMD who received either brolucizumab or faricimab found that both agents improved visual acuity after the loading phase, although brolucizumab led to faster visual gains, which equalized by month 4, and to greater reductions in central foveal and choroidal thickness than faricimab [14]. This difference was significant at month 2 but not at month 4, which suggests that, while faricimab is effective, brolucizumab may achieve faster anatomical improvement in the early phase of treatment, at the cost of a higher risk of adverse events.

Hoshino et al. investigated the effect of switching to brolucizumab in 33 patients with nAMD who showed persistent exudation despite treatment with faricimab [15]. This retrospective study found significant reductions in foveal and choroidal thickness, while visual acuity remained largely unchanged. Approximately one third of the eyes achieved a dry macula after the switch, but intraocular inflammation occurred in about 9% of cases. In another Japanese study, Takahashi et al. retrospectively analyzed 63 eyes of 60 patients with nAMD who were switched from brolucizumab to faricimab [16]. Of these, 38 continued with faricimab, while the remainder were switched back, received photodynamic therapy or paused treatment. In agreement with our findings, no significant difference in morphology or function was observed. nAMD is, however, known to differ between Asian and Caucasian patients [17, 18]. A substantial proportion of Asian patients have pachychoroid features, which may benefit more from Ang-2 inhibition, as shown in subgroup analyses, and more than 57% classify as polypoidal choroidal vasculopathy (PCV). These data can therefore not be compared directly with our Caucasian study population. Moreover, in the latter study faricimab was not administered at intervals shorter than 8 weeks for regulatory reasons, which may have resulted in an over-representation of poor anti-VEGF responders in the group that was switched back to brolucizumab.

To date, no randomised head-to-head trial has directly compared brolucizumab and faricimab. The real-world studies discussed above have, however, either compared the two agents directly or switched eyes between them [13–16]. Taken together, these reports indicate broadly comparable functional outcomes for the two agents, while the recognised association of brolucizumab with intraocular inflammation remains an important safety consideration. Placed in this broader context, our findings are consistent with an indirect comparison in which brolucizumab and faricimab offer similar functional stability but differ in their anatomical response and safety profile.

Beyond the direct brolucizumab-versus-faricimab reports discussed above, a broader body of real-world evidence has evaluated switching from aflibercept 2 mg to either agent in treatment-resistant nAMD, allowing an indirect comparison. Ota et al. switched 48 aflibercept-refractory eyes to brolucizumab and observed a marked reduction in central retinal thickness (298.9 μm to 241.9 μm, *p* < 0.001) with unchanged visual acuity (0.26 to 0.25 logMAR, *p* = 0.95); however, intraocular inflammation occurred in 14.6% of eyes and a comparable proportion had to be switched back because the treatment interval could not be extended to at least eight weeks [19]. On the faricimab side, Schneider et al. switched 50 aflibercept-resistant eyes to faricimab and reported an absence of fluid in 32% and a reduction of fluid in 84% of eyes, a significant decrease in central retinal thickness among responders, and only a marginal change in visual acuity, with no ocular or systemic safety events [20]. Taken together, these aflibercept-switch series suggest that both agents achieve meaningful anatomical improvement while preserving visual function in eyes that are otherwise difficult to treat, but that brolucizumab carries a higher risk of intraocular inflammation, whereas faricimab offers comparable anatomical benefit with a more favourable safety profile. This indirect comparison is consistent with the rationale for the brolucizumab-to-faricimab transition evaluated in the present study and with our observation that visual function remained stable after the switch.

In our study, all 31 eyes had received bevacizumab and aflibercept, and 8 of the 31 eyes had additionally received ranibizumab, before brolucizumab and the subsequent switch to faricimab. These 8 eyes therefore had a different prior anti-VEGF exposure from the remaining 23 eyes, which may have confounded the observed outcomes, particularly if the wash-out period before the transition to brolucizumab and then faricimab was not sufficiently long. This heterogeneity in prior treatment is acknowledged as a limitation.

A further consideration is that faricimab, like brolucizumab, is labelled for an initial loading phase of at least three doses before interval extension. In the present cohort, the morphological and functional status after three or more brolucizumab injections was set against the status after switching to faricimab, and the most pronounced anatomical change was observed after the first faricimab injection. Because these eyes had already received numerous anti-VEGF injections, a formal loading phase was not routinely repeated. The follow-up presented here (visits 1 to 3) shows that central retinal thickness in the poor-responder subgroup decreased again over the subsequent visits, supporting the view that an adequate loading phase should be considered when switching, especially in eyes with Type 1 MNV, which appeared more prone to renewed exudation and to require a higher injection frequency.

In addition, visual acuity was recorded as best-corrected Snellen acuity and converted to logMAR rather than measured with a standardized ETDRS protocol. Although the same approach was applied across all eyes, Snellen-based acuity is less precise and reproducible than protocol ETDRS testing, which should be borne in mind when interpreting the functional results.

According to the Safety Review Committee of the HAWK and HARRIER data, approximately 4.6% of eyes developed intraocular inflammation (IOI). No IOI occurred in our study; our patients had, however, a history of good tolerability of brolucizumab and therefore remained on this treatment until faricimab became available.

Retinal pigment epithelium tears were recorded in two eyes (6.5%) and macular atrophy in 12 eyes (38.7%). However, these were preexistent and cannot be attributed to either faricimab or brolucizumab.

Although the study is limited by its retrospective design and by the relatively small number of patients, the anatomical and functional stability observed in our study population provides useful insight into the early treatment dynamics following conversion from brolucizumab to faricimab. The follow-up period was short and, in two eyes, truncated by a subsequent switch back to brolucizumab; accordingly, the absence of serious adverse events up to the last recorded visit cannot be extrapolated to longer-term safety.

Two sources of bias follow specifically from the retrospective design. First, patients were not selected according to a protocol: the decision to switch, its timing and the interval chosen afterwards were made by the treating physician on clinical grounds, which introduces confounding by indication. The bias has a direction, in that eyes which had tolerated brolucizumab well over a long period were precisely those that remained on it until faricimab became available; this is the most likely reason why no IOI was observed and it means that our safety experience cannot be generalized to unselected eyes. Second, treatment intervals were not standardized, since the departmental rule left the decision in the individual eye to the treating physician; the CRT increase after the first faricimab injection therefore reflects both the change of agent and the interval extension applied to the fluid-free eyes, and the two cannot be separated in this design. Given the sample size and the number of exploratory comparisons, the association between Type 1 MNV and a poor response should be regarded as hypothesis-generating.

## Conclusions

In this retrospective case series of nAMD eyes switched from brolucizumab to faricimab, visual acuity remained stable and central retinal thickness was largely maintained across the overall cohort. However, a substantial proportion of previously inactive eyes developed renewed exudation and eyes with Type 1 MNV appeared particularly prone to a suboptimal response and to require a higher injection frequency. These findings suggest that, rather than relying on a single dose, an adequate faricimab loading phase and cautious interval extension should be considered when switching, especially in poor responders. Both agents remain viable treatment options, and the variability in individual responses underlines the need for personalized, response-guided treatment strategies. Larger prospective studies with longer follow-up and standardized loading are warranted to confirm these observations.

## Data Availability

The dataset generated and analysed during the current study is available in the Zenodo repository [21] (https://doi.org/10.5281/zenodo.21221774).

## Abbreviations

AMD: Age-related macular degeneration
Ang-2: Angiopoietin-2
BCVA: Best-corrected visual acuity
CRT: Central retinal thickness
EMA: European Medicines Agency
ETDRS: Early Treatment Diabetic Retinopathy Study
FDA: Food and Drug Administration
IOI: Intraocular inflammation
IRF: Intraretinal fluid
nAMD: Neovascular age-related macular degeneration
MNV: Macular neovascularization
OCT: Optical coherence tomography
PCV: Polypoidal choroidal vasculopathy
SRF: Subretinal fluid
VA: Visual acuity
VEGF: Vascular endothelial growth factor
